# The C-terminal region of human plasma fetuin-B is dispensable for the raised-elephant-trunk mechanism of inhibition of astacin metallopeptidases

**DOI:** 10.1038/s41598-019-51095-y

**Published:** 2019-10-11

**Authors:** Tibisay Guevara, Hagen Körschgen, Anna Cuppari, Carlo Schmitz, Michael Kuske, Irene Yiallouros, Julia Floehr, Willi Jahnen-Dechent, Walter Stöcker, F. Xavier Gomis-Rüth

**Affiliations:** 1 Proteolysis Lab, Department of Structural Biology, Molecular Biology Institute of Barcelona, CSIC, Barcelona Science Park, Helix Building, c/ Baldiri Reixac, 15-21, E-08028 Barcelona, Catalonia Spain; 2 Institute of Molecular Physiology, Cell and Matrix Biology, Johannes Gutenberg-University Mainz, Johann-Joachim-Becher-Weg 7, D-55128 Mainz, Germany; 3 Biointerface Laboratory, Helmholtz Institute for Biomedical Engineering, RWTH Aachen University Medical Faculty, Pauwelsstr. 30, D-52074 Aachen, Germany

**Keywords:** Proteases, X-ray crystallography

## Abstract

Human fetuin-B plays a key physiological role in human fertility through its inhibitory action on ovastacin, a member of the astacin family of metallopeptidases. The inhibitor consists of tandem cystatin-like domains (CY1 and CY2), which are connected by a linker containing a “CPDCP-trunk” and followed by a C-terminal region (CTR) void of regular secondary structure. Here, we solved the crystal structure of the complex of the inhibitor with archetypal astacin from crayfish, which is a useful model of human ovastacin. Two hairpins from CY2, the linker, and the tip of the “legumain-binding loop” of CY1 inhibit crayfish astacin following the “raised-elephant-trunk mechanism” recently reported for mouse fetuin-B. This inhibition is exerted by blocking active-site cleft sub-sites upstream and downstream of the catalytic zinc ion, but not those flanking the scissile bond. However, contrary to the mouse complex, which was obtained with fetuin-B nicked at a single site but otherwise intact, most of the CTR was proteolytically removed during crystallization of the human complex. Moreover, the two complexes present in the crystallographic asymmetric unit diverged in the relative arrangement of CY1 and CY2, while the two complexes found for the mouse complex crystal structure were equivalent. Biochemical studies *in vitro* confirmed the differential cleavage susceptibility of human and mouse fetuin-B in front of crayfish astacin and revealed that the cleaved human inhibitor blocks crayfish astacin and human meprin α and β only slightly less potently than the intact variant. Therefore, the CTR of animal fetuin-B orthologs may have a function in maintaining a particular relative orientation of CY1 and CY2 that nonetheless is dispensable for peptidase inhibition.

## Introduction

**Figure 1:**
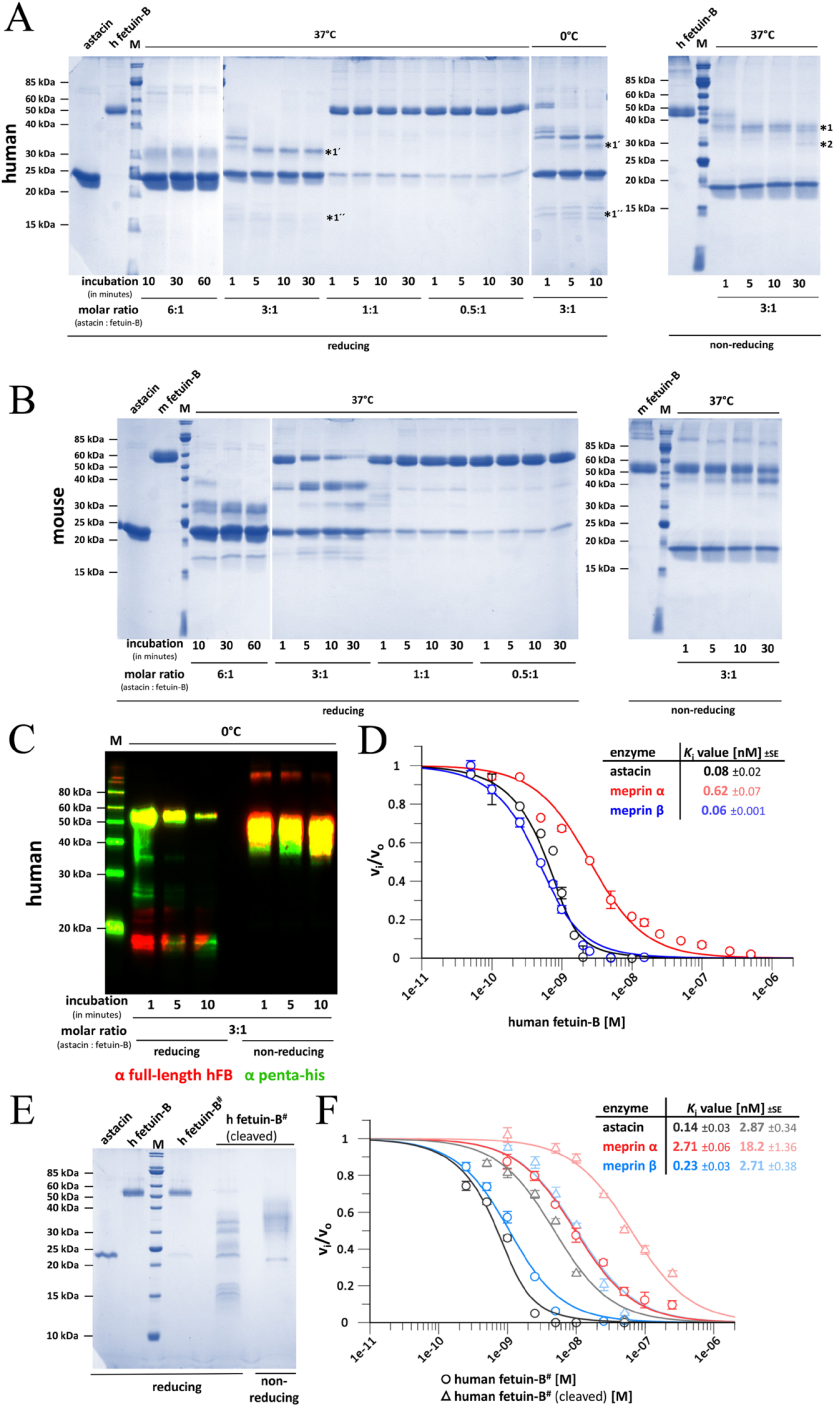
Digestion and inhibitory studies. Coomassie staining of hFB (**A**) and mFB (**B**) at 25 pmol/lane incubated with astacin and separated by reducing (*left*) and non-reducing (*right*) 14% SDS-PAGE. Bands subjected to N-terminal Edman degradation are pinpointed by asterisks and labeled 1, 1′, 1″ and 2, respectively. (**C**) Western-blot analysis of hFB (10 pmol/lane) incubated with astacin and separated by reducing and non-reducing 14% SDS-PAGE. Immunodetection was performed with anti-full-length hFB antibody (red) and anti-penta-His antibody (green). (**D**) Plot of fractional velocity (logarithmic scale) of peptidase inhibition by hFB. Enzyme and substrate concentrations were 1.0 nM and 170 mM for astacin, 1 nM and 25 mM for meprin α, and 0.5 nM and 25 mM for meprin β, respectively. Error bars indicate standard errors. The upper-right inset depicts the derived *K*_i_ values. (**E**) Coomassie staining of cleaved (3 µg/lane) and intact (1 µg/lane) hFB separated by 14% SDS-PAGE. Species pinpointed by a hashtag were treated with urea as described in the Materials and Methods section. (**F**) Plot of fractional velocity (logarithmic scale) of peptidase inhibition by urea-treated intact and cleaved hFB. Enzyme and substrate concentrations as in (D). Error bars indicate standard deviations. The upper-right inset indicates the derived *K*_i_ values of intact (*left*) and cleaved (*right*) hFB.

Metallopeptidases (MPs) are engaged in spatially and temporally regulated physiological processes including maturation, shedding and inactivation^1–3^. Owing to the irreversibility of peptide bond cleavage *in vivo*, MPs must be tautly restrained to prevent aberrant activity, which gives rise to dysfunction and disease. Control mechanisms include transcriptional regulation^4^, cellular and molecular compartmentalization^5^, zymogen-mediated latency^3,6^, and colocalization of specific or broad-spectrum protein inhibitors^7^. One of the many MP families described^7,8^ are the astacins, which were named for the eponymous digestive enzyme from the crayfish *Astacus astacus*^9–13^. Astacins have a common ~200-residue zinc-dependent catalytic domain (CD) whose architecture generally conforms to that of MPs of the metzincin clan^8,14–17^. Astacins are produced as zymogens with a pro-segment for latency upstream of the CD and a variable number of downstream domains (see Fig. 1 in^11^ for details), and six family members are found in humans: bone morphogenetic protein 1 and its major splice variant, mammalian tolloid, which are also known as procollagen C-proteases; tolloid-like proteins 1 and 2; meprin α and β; and ovastacin (see^11,12^ and http://degradome.uniovi.es/met.html ^18^). The first three MPs constitute the tolloid subgroup and process precursors of fibrillar procollagens, proteoglycans, laminins, and anchoring fibrils. They are thus important for extracellular-matrix assembly^11,19^. Tolloid astacins also cleave growth factors and their antagonists, which are crucial for dorso-ventral patterning during embryo gastrulation^20^. Next, meprins are membrane-bound proteins involved in tissue differentiation and pericellular signaling by processing of biologically active peptides, cytokines, chemokines, growth factors, and peptidase zymogens^21,22^. They also cleave components of the extracellular matrix such as the basal lamina, procollagens, and adhesion proteins^21,23,24^. Finally, ovastacin is a hatching enzyme expressed in the oocyte^25^. The enzyme cleaves the glycoprotein matrix surrounding the oocyte, dubbed the zona pellucida, after gamete fusion. This leads to rigidification of the matrix, blocks further sperm binding, and is essential for the survival of the developing embryo^25–27^.

Small astacins, but not large multidomain forms such as the meprins, are inhibited by the non-specific pan-peptidase inhibitor α_2_-macroglobulin^28–30^. Specific inhibition of tolloid astacins has been reported for *Xenopus laevis* sizzled/ogon^31,32^. By contrast, meprins, crayfish astacin, nephrosin from cyprinid fishes, and ovastacin are strongly inhibited by fetuin-B forms from mammals, which are strictly selective for astacins^33–36^, and by fish fetuin, which acts as the physiological antagonist of nephrosin^37^. By blocking ovastacin, fetuin-B prevents premature hardening of the zona pellucida and maintains female fertility^26,33,34^. Fetuin-B belongs to the I25 family of peptidase inhibitors according to the MEROPS database of peptidases and inhibitors (www.ebi.ac.uk/merops)^7^. The archetype of this family is chicken egg-white cystatin (ovocystatin), a 116-residue reversible inhibitor specific for cysteine peptidases^38,39^. Within the family, fetuins are type-3 cystatins (subfamily I25C), which include glycosylated proteins with two or three cystatin-like modules^40,41^. Recent crystal structures of the mouse ortholog (mFB), isolated and in complex with crayfish astacin^36^, have revealed that the inhibitor consists of the tandem cystatin-type modules 1 and 2 (CY1 and CY2), which are united by a linker (LNK) with a “CPDCP-trunk” and followed by a C-terminal region (CTR). The inhibitor blocks the active-site cleft of the MP following a novel “raised-elephant-trunk” mechanism^36^.

To complement these studies, we here report the crystal structure of the complex between the human ortholog of fetuin-B (hFB), which is the physiologically relevant species for studying human fertility^42^, and 202-residue mature crayfish astacin, which is a useful model for the 197-residue catalytic domain of human ovastacin (35% sequence identity; 48% similarity; see also^35^). These studies revealed unexpected differences with mFB in terms of proteolytic susceptibility and the spatial arrangement of the cystatin domains, which enabled us to identify dispensable structural elements for inhibition. We verified these structural findings by means of biochemical studies with crayfish astacin and human meprins *in vitro*.

## Results and Discussion

### Crystallization and proteolytic susceptibility of human fetuin-B in the presence of crayfish astacin

We have previously reported the structure of isolated mFB, which was recombinantly produced in mammalian cells and processed with endoglycosidase H^36^. This treatment clipped down the *N*-glycans attached to *N*^40^ and *N*^139^ (mFB amino-acid numbering in superscript and italics according to UniProt entry [UP] Q9QXC1) to single *N*-acetylglucosamine moieties. In particular, the sugar attached to *N*^40^ participates in a packing contact that is crucial for crystal formation. In contrast, no crystals have been obtained with protein heterologously produced in insect cells, which attach different glycans. However, for the astacin·mFB complex crystal structure, protein from insect cells has been successfully employed in the presence of MP excess^36^. In this study, hFB was recombinantly produced and purified from mammalian cells, and crystallization of the astacin·hFB complex was also only successful with peptidase excess. SDS-PAGE, Western blotting, and N-terminal Edman degradation of the complex in solution revealed that most of the CTR was removed through cleavages at positions G^302^-S^303^ (hFB residue numbering in superscript according to UP Q9UGM5), E^322^-A^323^ and somewhere upstream within the CTR to yield species migrating according to ~30–35 kDa instead of the ~50 kDa of intact hFB (Fig. 1A,C). Cleaved forms were isolated by astacin affinity chromatography for comparative inhibitory studies (see Fig. 1E and below). In contrast to hFB, mFB proved more resistant to astacin degradation under equivalent conditions (Fig. 1B). This is consistent with the aforementioned crystallization experiments^36^, which revealed that peptidase excess cleaves mFB at bond *S*^296^*-S*^297^ within the CTR of the otherwise intact molecule^36^. The structure of the mouse complex has shown that the CTR is an irregularly folded domain that is partially disordered and devoid of regular secondary structure, which explains its proteolytical susceptibility.

Astacin·hFB crystals diffracted to a similar resolution as those of the mouse complex (3.0 Å *vs*. 3.1 Å) and likewise contained two enzyme·inhibitor complexes per asymmetric unit (A·B and C·D). However, they belonged to a different space group (P6_1_ *vs*. P2_1_2_1_2_1_; compare Table 1 and Table 1 in^36^), had different cell constants, and contained significantly more solvent (57% *vs*. 47%).

**Table 1:** Crystallographic data.

Dataset	Crayfish Astacin·Human Fetuin-B
Space group	P6_1_
Cell constants (a, b, c, in Å)	90.7, 90.7, 283.9
Wavelength (Å)	1.0032
No. of measurements/unique reflections	240,701/26,287
Resolution range (Å)	78.5–3.00 (3.18–3.00)^a^
Completeness (%)	99.7 (98.9)
R_merge_	0.184 (1.565)
R_meas_	0.195 (1.660)
CC^1/2^	0.996 (0.626)
Average intensity	11.1 (1.7)
B-Factor (Wilson) (Å^2^)	70.5
Aver. multiplicity	9.2 (8.9)
No. of reflections used in refinement [in test set]	25,577 [708]
Crystallographic R_factor_/free R_factor_	0.185/0.247
Correlation coefficient *F*_obs_-*F*_calc_ [test set]	0.944 [0.890]
No. of protein residue/atoms/solvent molecules/	897/6,832/31/
covalent ligands/	7 NAG, 2 FUC, 2 BMA^b^/
non-covalent ligands	2 Zn^2+^
*Rmsd* from target values	
bonds (Å)/angles (°)	0.009/1.06
Average B-factors (Å^2^) (overall//mol. A/B/C/D)	82.4//71.2/92.4/70.9/91.7
All-atom contacts and geometry analysis^c^	
Protein residues	
in favored regions/outliers/all residues	809 (95.2%)/5/850
with outlying rotamers/bonds/angles/chirality/torsion	33 (4.4%)/0/0/0/0
All-atom clashscore	3.0

^a^Data processing values in round brackets are for the outermost resolution shell. ^b^NAG, *N*-acetyl-D-glucosamine; FUC, α-1-fucose; and BMA, β-D-mannose. ^c^According to the wwPDB X-ray Structure Validation Service.

### Structure of cleaved human fetuin-B

**Figure 2:**
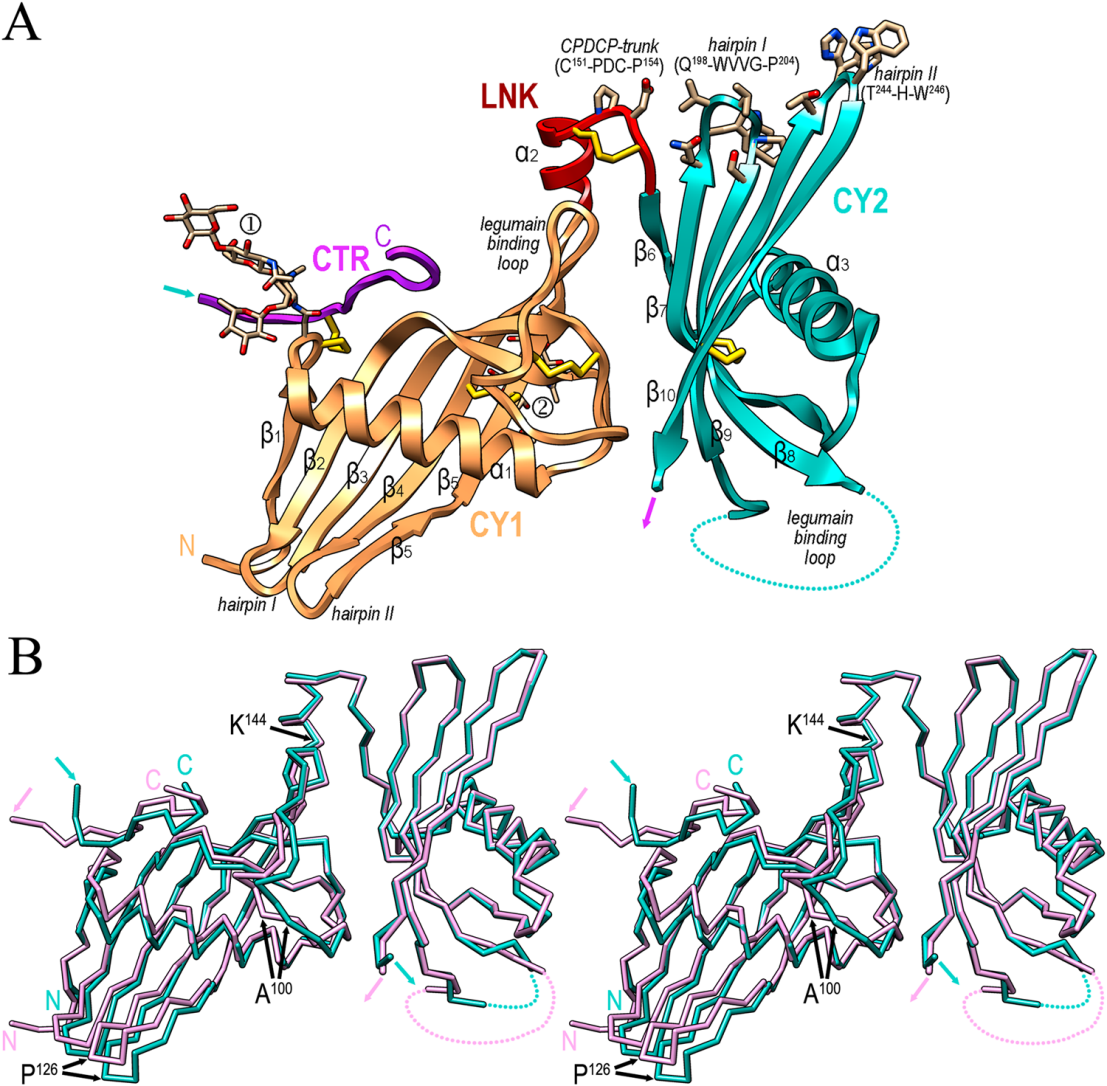
The cleaved human fetuin-B structure. (**A**) Ribbon-type plot of cleaved hFB (molecule B), which consists of domains CY1 (in orange) and CY2 (turquoise), linker LNK with the CPDCP-trunk (red) and a short fragment of the CTR (purple). Inter- and intra-domain disulfides are indicated as yellow sticks (for the respective residue numbers, see Fig. 4A). N-linked glycans are attached to N^37^ (①) and N^136^ (②). The regular secondary structure elements are labeled (β1-β10, α1-α3; for the respective spanning residues see Fig. 4A). The legumain-binding loops are labelled, as well as hairpins I and II of each cystatin domain. Relevant residues from the CPDCP-trunk and the tips of CY2 hairpin I and II are shown for their side chains. Residue H^245^ is in double occupancy in the A·B complex. The LBL of CY2 is partially disordered (dotted line). After the last visible residue of strand β10 (magenta arrow), the polypeptide chain only becomes defined again at CTR residues R^364^/T^365^ (turquoise arrow). (**B**) Superposition of hFB molecules B (in plum) and D (turquoise) reveals a hinge motion about K^144^ and significant variations around A^100^ and P^126^. Turquoise and plum arrows pinpoint the last CY2 residues and the first CTR residues visible in the final Fourier map.

The structure spans domains CY1 (A^25^/N^27^-S^143^) and CY2 (S^156^-E^258^/S^259^), which are connected by the linking segment LNK (K^144^-P^155^), plus a small fragment of the CTR (R^364^/T^365^-A^375^/S^376^) (Fig. 2A). Characteristic of cystatin-type domains, CY1 and CY2 consist of an antiparallel five-stranded curled β-sheet of up-and-down connectivity (strands β1-β5 in CY1 and β6-β10 in CY2) and a perpendicular α-helix (α1 in CY1 and α3 in CY2) inserted between the first two strands. The second/third and fourth/fifth strands of each sheet are joined by short connections (hairpins I and II, respectively), while the third and fourth strands are connected by long “legumain-binding loops” (LBLs)^36,43^ of 21 and 19 residues in CY1 and CY2, respectively. In the latter domain, the LBL is disordered for C^216^-S^228^. In contrast, in CY1 it is well-defined and creates a hydrophobic pillow at its most exposed segment spanning I^108^-F^110^. Residing on this pillow, LNK comprises helix α2 and the CPDCP-trunk (C^151^-P^155^), which protrudes from the molecular surface (Fig. 2A). Finally, a 12-residue fragment of the CTR is attached to CY1 through disulfide C^36^-C^368^. Further disulfides are found in the LBLs (C^93^-C^104^ in CY1 and, probably, C^216^-C^224^ in CY2), as well as between β4 and β5 of CY1 (C^117^-C^137^) and β9 and β10 of CY2 (C^237^-C^254^). A last disulfide (C^151^-C^154^) tightly crosslinks LNK to provide rigidity to the CPDCP-trunk. Within CY1, two *N*-glycan chains are attached to N^37^ and N^136^, respectively (Fig. 2A).

The two hFB moieties in the asymmetric unit (B and D) superpose with an *rmsd* of 1.1 Å. While the respective CY2 and LNK moieties fit well, the CY1 domains are rotated by ~5° around K^144^Cα, which leads to a displacement of maximally ~4.5 Å (at P^126^Cα) (Fig. 2B). In addition to this rigid-body displacement, which in general keeps the same conformation in both CY1 domains, it is remarkable that significant rearrangement is found in segment R^97^-M^106^ within the LBL, which is displaced by ~5 Å maximally (at A^100^Cα).

### Inhibition of crayfish astacin by human fetuin-B

Crayfish astacin is a bipartite molecule of 202 residues consisting of two equally large upper and lower sub-domains (USD and LSD), which form an extended, deep active-site cleft at their interface^9,10,44^. The cleft harbors the catalytic zinc ion, which is bound by three histidines from a zinc-binding consensus sequence (H_92_-EXXHXXGXX-H_102_; mature residue numbering of astacin in subscript; for numbering of the preproprotein according to UP P07584, add 49), which further contains the general base/acid for catalysis (E_93_) and is a hallmark of the astacins^11,12^ and other metzincin MPs^8,14,17^.

**Figure 3:**
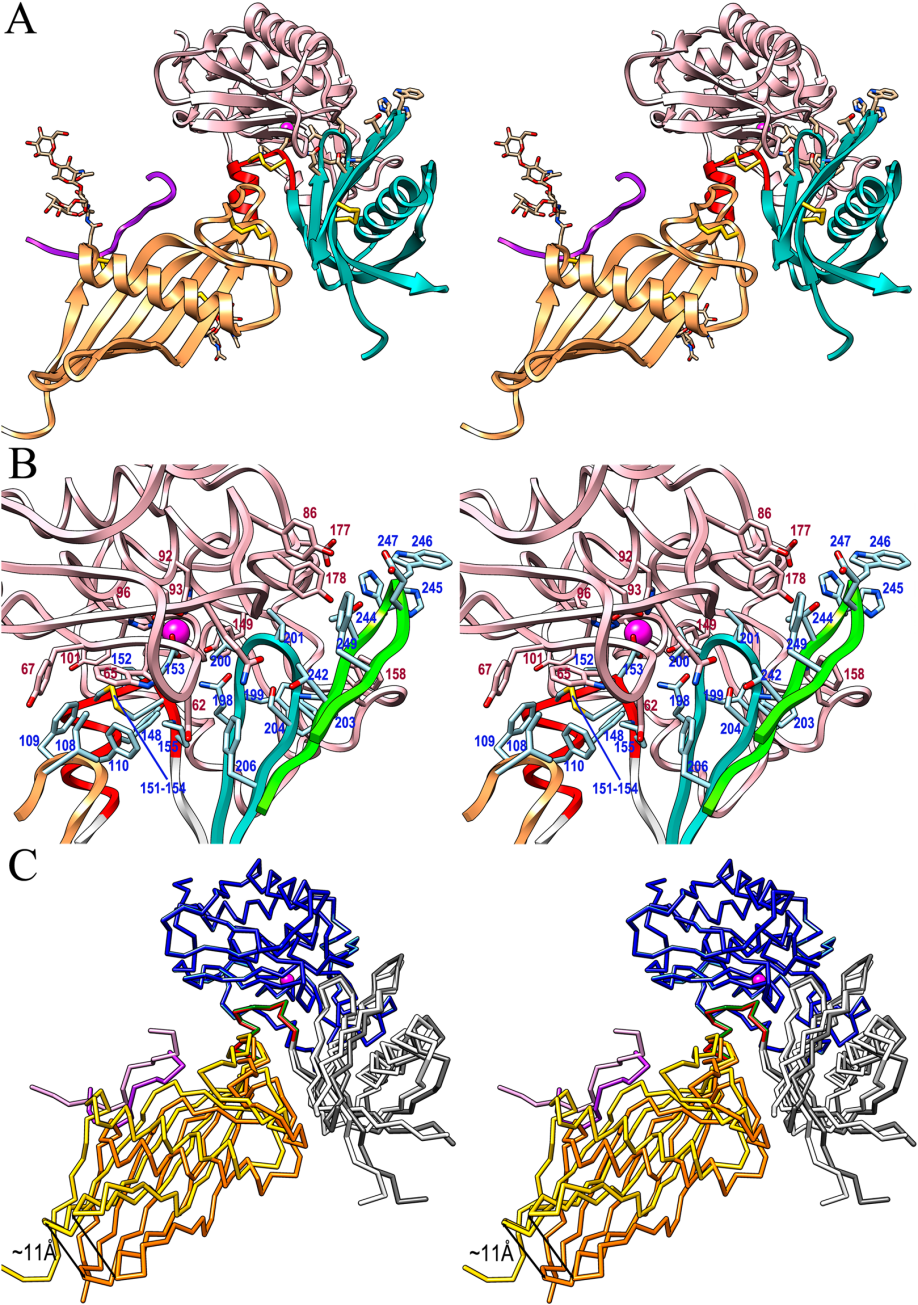
Structure of the astacin complex with cleaved human fetuin-B. (**A**) Ribbon-type plot in cross-eye stereo of the crayfish astacin·hFB complex A·B. The inhibitor is colored as in Fig. 2A, astacin is in salmon, and the catalytic zinc ion is shown as a magenta sphere for reference. The orientation results from that of Fig. 2A after a horizontal downward rotation of ~40°. This corresponds to a ~90°-rotation downward with respect to the standard orientation of astacin (see^49^). (**B**) Close-up view of (A) depicting the principal residues engaged in complex formation of astacin (pink ribbon and carbons; residue numbers in dark red) and hFB (tip of CY1 LBL as an orange ribbon, LNK as a red ribbon, CY2 hairpin I as a turquoise ribbon, and CY2 hairpin II as a green ribbon) with carbons in pale blue (residue numbers in blue). (**C**) Superposition of the enzyme moieties of the two astacin·hFB complexes A·B (astacin in sky blue, CY1 in yellow, LNK in red, CY2 in white, and CTR in pink) and C·D (astacin in blue, CY1 in orange, LNK in green, CY2 in gray, and CTR in purple) reveals flexibility of domain CY1.

In the complex, the hFB moiety inserts like a chock into the active-site cleft of astacin through contacts made by the LNK, hairpins I and II of CY2, and the tip of the LBL of CY1 (Fig. 3A,B). This causes the cleft of both MP protomers in the asymmetric unit (A and C) to slightly open, triggered by a ~7°-rotation of the LSD around a horizontal axis traversing F_100_ and P_176_, which causes a maximal displacement of ~3 Å (at S_123_Cα). Owing to the slight differences between hFB moieties B and D (see previous section), the A·B and C·D complexes also vary. Superposition of the respective MP moieties reveals that while the chock elements of the hFBs coincide, the cleft-distal parts of the inhibitors are rotated relative to each other by ~12° about a horizontal axis traversing T^150^ and V^201^, which causes a displacement of ~11 Å maximally (at P^126^; see Fig. 3C). This is reminiscent of the complex between the otherwise unrelated MP thermolysin from *Bacillus thermoproteolyticus* and its specific inhibitor IMPI from the greater wax moth *Galleria mellonella*^45^. The latter likewise follows a wedge-like inhibition mechanism involving a few structural elements on the inhibitor surface. In the two complexes in the asymmetric unit, the inhibitor moieties are rotated by ~5° relative to each other by visual inspection.

In the hFB·astacin complex A·B (Fig. 3A,B), the interface spans 1,245 Å^2^, with a theoretical solvation energy gain on complex formation (Δ^i^G according to^46^) of −20.1 kcal/mol and a significant complexation significance score (CSS according to^46^) of 0.50. The interface involves 35 residues of hFB and 41 of astacin, which make 14 hydrogen bonds, two ionic interactions, and hydrophobic interactions between 27 pairs of residues (see Table 2). Astacin segments involved in the complex are S_62_-V_68_, Q_80_-H_102_, S_126_-Y_133_, and Y_149_-W_158_. Participating hFB regions include I^108^-E^111^ from the LBL of CY1 and Y^148^-P^155^ from helix α2 plus the downstream CPDCP-trunk, which from M^149^ to D^153^ runs along the non-primed side of the cleft in the direction and orientation of a substrate (Fig. 3B). In particular, D^153^ binds the catalytic zinc of astacin according to an aspartate-switch mechanism, as well as the general base/acid E_93_ and Y_149_. After D^153^, the polypeptide performs a ~90°-kink outward and leaves the cleft, which explains why fetuin-B is not cleaved at bond D^153^-C^154^. This contrasts with standard-mechanism inhibitors^47^ of MPs, such as the aforementioned IMPI from which a reactive-site loop is inserted like a substrate across the entire cleft of thermolysin. Thus, the reactive-site bond that links residues in sub-site positions **S**_**1**_ and **S**_**1**_**’** (for peptidase active-site cleft sub-site nomenclature, see^48,49^) is cleaved^45^.

**Table 2:** Interactions at the interface between astacin and human fetuin-B.

Hydrogen bonds	Hydrophobic interactions
Υ^148^Οη – N_127_O 3.3/3.2 Å	
Υ^148^Οη – D_129_O 3.8/3.5 Å	
M^149^O – Y_101_Oη 2.9/2.9 Å	I^108^ – W_65_F^109^ – W_65_
T^150^O – V_68_N 3.0/2.9 Å	F^109^ – Y_67_F^110^ – W_65_
C^151^Sγ – S_66_O 3.4/3.5 Å	Y^148^ – N_127_M^149^ – Y_133_ (A·B)
D^153^Oδ2 – E_93_Oε1 3.0/3.4 Å	M^149^ – T_132_C^151^ – W_65_
D^153^Oδ2 – E_93_Oε2 2.8/2.9 Å	P^152^ – V_68_P^152^ – H_96_
D^153^Oδ1 – Y_149_Oη 2.7/2.6 Å	P^152^ – Y_101_P^152^ – H_102_
Q^198^Nε2 – C_64_O 3.0/2.8 Å	C^154^ – W_65_P^155^ – W_65_
Q^198^Oε1 – C_64_N 3.4/3.2 Å	W^199^ – S_153_W^199^ – F_154_
V^201^Ο – Y_177_Οη 3.7/ – Å	W^199^ – W_158_V^200^ – C_64_
S^240^Oγ – Q_80_Nε2 3.9/ – Å	V^201^ – C_64_V^201^ – Y_177_
T^242^Oγ1 – Q_80_Οε1 3.2/3.4 Å	P^203^ – W_158_F^206^ – S_62_
H^245^Nδ1 – Υ_177_Οη 2.9/3.3 Å	F^206^ – G_63_T^244^ – W_158_
H^245^Nδ1 – D_178_Οδ1 – / 3.9 Å	H^245^ – W_158_ (A·B)W^246^ – Y_177_ (C·D)
Ionic interactions	F^249^ – N_82_F^249^ – G_83_
D^153^Oδ1 – Zn_999_ 2.2/2.2 Å	
D^153^Oδ2 – Zn_999_ 2.2/2.2 Å	

The first residue/atom belongs to fetuin-B, the second to astacin. The two distances indicated for electrostatic interactions correspond to the A·B and C·D complexes, respectively. Hydrophobic interactions are for both the A·B and C·D complexes, if not otherwise stated in parenthesis.

Assisting the CY1 LBL and the CPDCP-trunk, the tip of hairpin I of CY2 spanning Q^198^-F^206^ blocks sub-sites on the primed side of the cleft (Fig. 3B). This hairpin contains segment Q^198^-WVVG-P^203^, which conforms to the hallmark sequence motif of inhibitory fetuins (QWVXGP^36^), and V^200^ and V^201^ nestle into sub-sites **S**_**2**_**’** and **S**_**3**_**’**, respectively. Finally, the tip of CY2 hairpin II (S^240^-F^249^) further contributes to the complex by blocking outmost cleft sub-sites and attaching to the right surface of the enzyme (Fig. 3B). Overall, these interactions conform to the raised-elephant-trunk mechanism of astacin inhibition previously described in detail for mFB^36^. Thus, the present structure suggests that the excised CTR is not required for inhibition.

As expected from a comparison of the isolated inhibitor moieties (see previous section), superposition of the peptidases of the two complexes in the asymmetric unit reveals that the segments engaged in inhibition (tip of CY1 LBL, LNK with CPDCP-trunk and hairpins I and II of CY2), as well as the rest of CY2 (Fig. 3C), appear well-aligned. Hence, the aforementioned interactions at the A·B enzyme·inhibitor interface are also found in the C·D complex with just a couple of exceptions (Table 2). In contrast, the rest of CY1 with the attached CTR fragment significantly deviates in both complexes owing to a ~12°-rotation about K^144^, which causes a displacement of ~11 Å maximally, at the cleft-distal edge around G^77^Cα and S^127^Cα (Fig. 3C). In line with these differences, in complex C·D the interface spans 1,421 Å^2^, with a Δ^i^G of −20.4 kcal/mol and a CSS of 0.43. A total of 40 residues of hFB and 43 of astacin participate in the interface through 13 hydrogen bonds, two ionic interactions and hydrophobic interactions between 26 pairs of residues (see Table 2). Taken together, these findings support an ancillary role for CY1 in inhibition and, possibly, a tethering role for the missing CTR to fix the relative orientation of CY1 and CY2, although crystal packing may also play a role.

### Inhibitory studies *in vitro*

To assess the role of the CTR and the arrangement between CY1 and CY2 in peptidase inhibition, we first determined the apparent inhibition constant *K*_i_ of intact hFB in front of crayfish astacin and human meprin α and β as a control of the generally valid inhibition of astacins by hFB, and found values in the subnanomolar range (Fig. 1D). These values were indicative of very potent inhibition and compared well with those of mFB (see Fig. 3 in^36^). Next, we isolated astacin-cleaved hFB (see above), which entailed treatment with urea to dissociate the inhibitor from the peptidase, and determined its *K*_i_ values in front of the aforementioned enzymes. As a control, we also determined these parameters for intact hFB subjected to the same chaotropic treatment (Fig. 1F). We found that urea slightly decreased the inhibitory power of intact hFB.However, the cleaved inhibitor still evinced potent inhibition with *K*_i_ values in the nanomolar range (Fig. 1D,F). We conclude that the CTR and the relative orientation of CY1 and CY2 play a negligible role in the inhibition of astacin MPs.

### Comparison with the mouse fetuin-B·crayfish astacin complex

**Figure 4:**
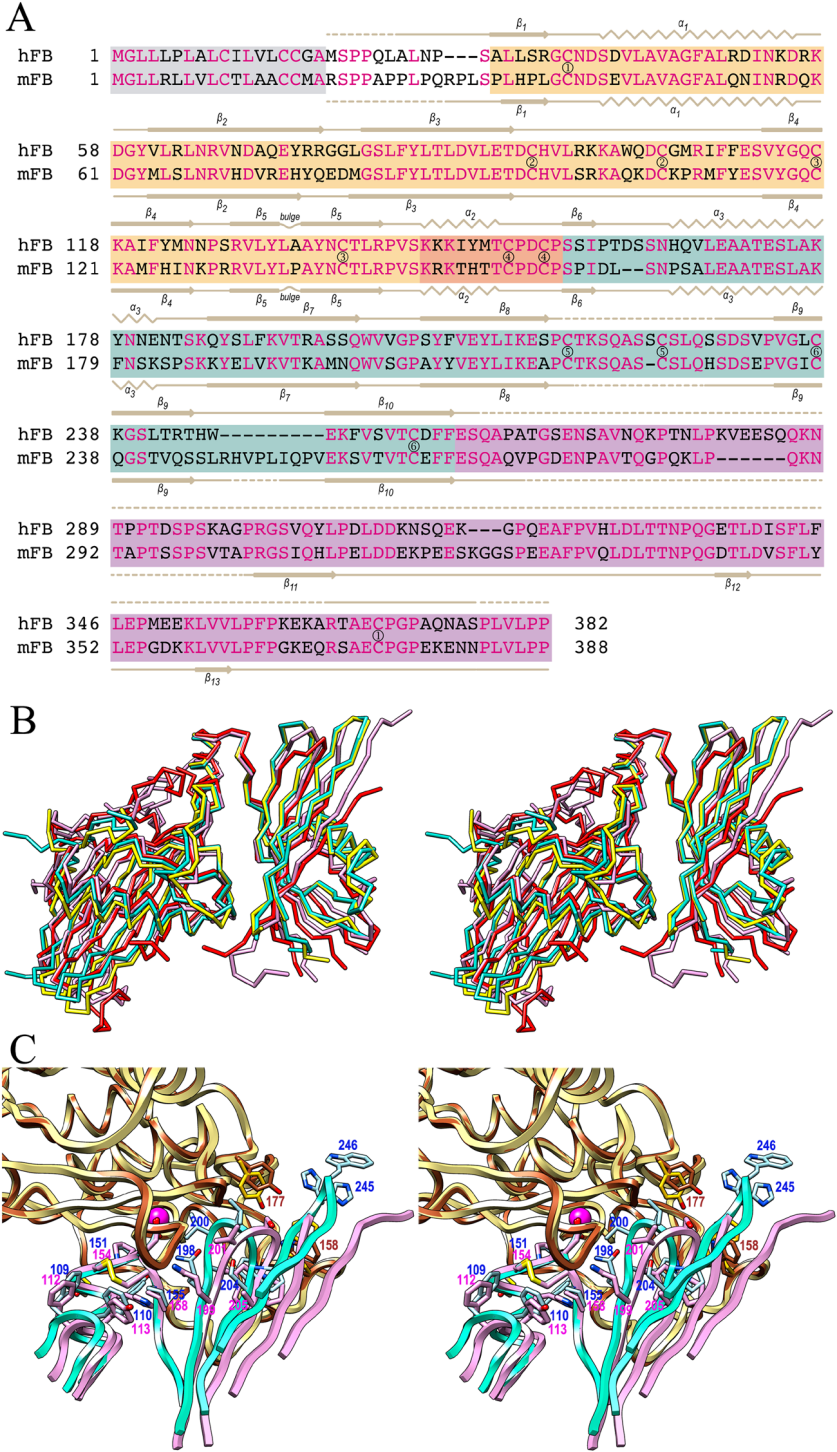
Comparison of human and mouse fetuin-B. (**A**) Structure-assisted sequence alignment of the human and mouse orthologs, identical residues (62%) are in magenta. Residues of CY1, LNK, CY2, CTR are over orange, salmon, turquoise and purple backgrounds, respectively. Predicted signal peptides are over a gray background. The lines above and below the alignment indicate missing residues in the astacin-complex structures (dashed lines), as well as β-strands (arrows labelled β1-β10) and α-helices (zigzags labelled α1-α3) of human and mouse fetuin-B, respectively. Residues connected by disulfide bonds are denoted by encircled numbers (①–⑥), C^216^-C^224^ (hFB) and *C*^217^-*C*^224^ (mFB) are tentatively assigned as the respective LBL segments are disordered. (**B**) Superposition of the two structures of hFB (Cα-traces of protomers B and D in turquoise and yellow, respectively), unbound mFB (Cα-trace in red), and astacin-bound mFB (Cα-trace in plum). The orientation corresponds to that of Fig. 2B. (**C**) Close-up view of (**B**) at the respective interfaces of hFB (ribbons in turquoise and carbons in light blue; residue labels in dark blue) and mFB (ribbons and carbons in plum; residue labels in magenta) with astacin (ribbons and carbons in brown and yellow, respectively; brown labels). The orientation corresponds to that of Fig. 3B.

Despite the lack of almost the entire CTR in the human inhibitor, but consistently with a sequence identity of 67% based on the CY1-LNK-CY2 domains involved in enzyme·inhibitor contact (Fig. 4A), the general inhibition modes of CTR-nicked mFB and CTR-depleted hFB are very similar and generally encompass the same segments, which have been outlined above and extensively described in^36^. However, significant differences can be found on detailed comparison.

First, in contrast with hFB, the two astacin·mFB complexes within the asymmetric unit evince only minor variations, mainly in the CTRs, as reflected by an *rmsd* of 0.85 Å. In the mouse complex, the complex interface spans 1,059 Å^2^ and gives a Δ^i^G of −10.8 kcal/mol and a CSS of 0.33. The interface involves 33 residues of fetuin-B and 38 of astacin, which perform ten hydrogen bonds, two ionic interactions and 20 hydrophobic interactions^36^. All these values are significantly lower than those for the two astacin·hFB interfaces (see above and Table 2), thus pointing to looser interaction in the mouse complex. This difference in inhibitory action is apparently reflected by the disparate serum concentrations of mFB (156 ± 3 µg/ml) and hFB (5 ± 1 µg/ml)^50^.

Second, superposition of the entire complexes reveals *rmsd* values of 1.9 Å and 2.2 Å for mFB compared with hFB complexes A·B and C·D, respectively. If the respective peptidase moieties are superposed, the leftmost part of the chock comprising LNK and the LBL tip of CY1 (I^108^-F^110^ in hFB and *M*^111^*-Y*^113^ in mFB) neatly coincide for their main chains and most interactions are equivalent, including those of the CPDCP-trunk aspartate (D^153^ in hFB and *D*^156^ in mFB) (Fig. 4B). However, the rest of the fetuin-B moieties diverge substantially. Within the coil connecting α3 and β7 of CY2, deviations of ~10 Å and ~8 Å are detected between mFB *K*^186^Cα and topologically equivalent K^185^Cα from hFB molecules B and D, respectively. Similarly, mFB *R*^130^Cα and equivalent S^127^Cα at the tip of CY1 hairpin II are ~7 Å apart in both hFB molecules B and D. Even within the better fitting parts of the complexes, the first two residues of the second turn of LNK helix α2 of hFB (Y^148^-M^149^) penetrate the non-primed side of the cleft of astacin more deeply than the equivalent pair of mFB, *H*^151^-*T*^152^ (Fig. 4B).

Third, both hairpins I and II of CY2 on the primed side of the cleft significantly diverge. While the former has the same length in both fetuins and likewise comprises the hallmark motif QWVXGP (*Q*^199^*-WVSG-P*^204^ in mFB), in hFB it intrudes the astacin cleft more deeply owing to a relative ~90°-rotation of the Cα-C bond of the tryptophan. This causes the side chain of the downstream valine to enter the **S**_**1**_**’** sub-site while in mFB it merely performs a hydrophobic interaction with disulfide C_64_-C_84_ on the top of the active-site cleft (Fig. 4B). In addition, hairpin II is eight residues shorter in hFB and, while slightly flexible at its tip (H^245^-R^246^), it is clearly resolved in the final Fourier map. In contrast, it is disordered and untraceable for four and six residues in the two mFB complexes. Thus, interactions of hFB with Y_177_-D_178_ and W_158_ of astacin that could not be detected in the mouse complex were defined here (Fig. 4B and Table 2). Interestingly, mFB stands alone with its long CY2 hairpin II, which uniquely contains seven-to-nine residues more than fetuin-Bs from other animals, including the evolutionarily closely related naked mole rat (see Suppl. Fig. 3 in^36^). This suggests that the other orthologs will probably have a structured CY2 hairpin II and bind astacins more similarly to hFB than to mFB.

## Conclusions

We crystallized the complex between hFB and crayfish astacin in the presence of an excess of the MP, which proteolytically removed most of the CTR but retained the complex-formation and inhibitory capacities of the fetuin. As found for the mouse ortholog, hFB blocked astacin following a raised-elephant-trunk mechanism by inserting like a chock into the active-site cleft but sparing sub-sites **S**_**1**_ and **S**_**1**_**’**. This explains why hFB was not cleaved at the inhibitory loops in contrast with standard-mechanism inhibitors.

The two astacin·hFB complexes in the asymmetric unit evinced significant differences in the relative arrangement of CY1 and CY2. This differs from the mouse complex, which was only nicked at the CTR upon incubation with an equivalent excess of astacin and for which the two complexes in the asymmetric unit were similar. This indicates that the proteolytic susceptibility of the human and mouse fetuin-B orthologs vary despite close relatedness based on high sequence identity. In the mouse complex, the interaction surface and the theoretical solvation energy gain on complex formation were significantly smaller than in the human CTR-depleted complex, which points to a looser enzyme·inhibitor interaction of the intact mouse inhibitor.

Biochemical assays *in vitro* revealed that intact hFB and mFB inhibited crayfish astacin and human meprin α and β with comparable potency, with *K*_i_ values in the subnanomolar range. This suggests similar inhibition mechanisms for both full-length orthologs and indicates that the unstructured CTR has a function in structural maintenance of a particular relative disposition between CY1 and CY2 in fetuins. This fixed arrangement probably limits the adaptive capacity of the intact inhibitor to produce an optimal interaction surface with the target MP as large as that of the cleaved form. Notably, the *K*_i_ values of cleaved hFB in front of crayfish astacin and meprins α and β were still in the nanomolar range.

## Materials and Methods

### Protein production and purification

Protein hFB with a C-terminal hexahistidine-tag, thus spanning residues C^16^-P^382^-IEGRHHHHHH, was expressed and secreted to the extracellular medium by mammalian ExpiCHO-S cells according to the manufacturer’s instructions (ThermoFisher Scientific) as previously reported^35^. Human meprin β was obtained as a zymogen as described^51^ and activated with trypsin^52^. Protein mFB with a C-terminal hexahistidine-tag was cloned in vector pFASTBac1 and expressed in baculovirus-transduced High Five cells as described for meprin^35,51,53^. Human meprin α was produced as described^53^. All proteins used for crystallization were purified by nickel-nitrilotriacetic-affinity and size-exclusion chromatography steps as published^33,35^. Astacin was purified from the digestive fluid of the European freshwater crayfish *Astacus astacus* as reported^10^.

### Crystallization and diffraction data collection

Crystallization assays were set up following the sitting-drop vapor diffusion method at the joint IBMB/IRB Automated Crystallography Platform of Barcelona Science Park. A Tecan robot (Tecan Trading) was used to prepare reservoir solutions, and a Cartesian Microsys 4000 XL robot (Genomic Solutions) or a Phoenix nanodrop robot (Art Robbins Instruments) dispensed nanocrystallization drops on 96 × 2-well Swissci Polystyrene MRC Crystallization Plates (Molecular Dimensions). Plates were stored at 4 °C or 20 °C in thermostatic crystal farms (Bruker AXS). The astacin·hFB complex only crystallized after incubating the inhibitor (at 7.5 mg/mL) with six-fold molar excess of the peptidase in 10 mM Tris·HCl, 140 mM sodium chloride, pH 6.8. Crystals were obtained at 20 °C in 200 nL:100 nL drops with protein complex solution and 20% [w/v] polyethylene glycol 3,350, 0.2 M sodium tartrate dibasic as reservoir solution. Crystals were cryo-protected through quick immersion in drops containing reservoir solution implemented with 20% [v/v] glycerol and flash vitrified in liquid nitrogen. They were mounted on a MD2M diffractometer (Maatel) by a CATS Automatic Sample Changer (Irelec) at beam line XALOC^54^ of the ALBA synchrotron in Cerdanyola (Catalonia, Spain) and kept at 100 K in the cryostream of an Oxford Cryosystems 700 series. Diffraction data was collected on a Pilatus 6 M pixel detector (Dectris) and processed with programs XDS^55^ and XSCALE^56^, and converted to the MTZ-format used by the CCP4 suite of programs^57^ with XDSCONV. Crystals belonged to space group P6_1_, contained two peptidase·inhibitor complexes per asymmetric unit (molecules A·B and C·D, respectively), and diffracted to 3.0 Å resolution (see Table 1 for data processing statistics).

### Structure solution and refinement

The structure of the astacin·hFB complex was solved by maximum-likelihood-scored molecular replacement with the PHASER program^58^. Initial searches with the entire astacin·mFB complex (PDB 6HT9^36^) were unsuccessful. Subsequent searches with the separate astacin and mFB moieties yielded chemically reasonable solutions upon visual inspection with a log-likelihood gain of 2,714 at final Eulerian angles and fractional cell coordinates (α, β, γ, x, y, z) 8.4, 120.3, 180.2, −0.274, 0.249, −0.003 and 178.5, 121.1, 159.3, 0.172, −0.239, −0.026 for astacin; and 359.7, 108.4, 176.7, −0.248, 0.331, −0.045 and 234.8, 111.1, 165.6, 0.453, 0.149, 0.109 for fetuin-B. However, this solution showed severe clashes between CY1 domains. Thus, a final search was performed with astacin *plus* the CY2 domain and LNK of mFB (*S*^146^*-E*^267^) of the latter solution and the mFB CY1 domain (*R*^29^-*V*^145^) separately. These calculations gave two solutions at 5.0, 0.3, 355.0, 0.000, 0.000, 0.001 and 270.1, 16.4, 270.5, 0.883, 0.985, −0.002 for the first searching model and two more for the second searching model at 291.6, 1.0, 128.0, −0.001, −0.008, 0.166 and 270.5, 7.3, 328.3, 0.837, 0.893, 0.162. The crystal packing was acceptable, and the log-likelihood gain was 2,508. Thereafter, automated model building was used to input the sequence of hFB and density modification was carried out with twofold averaging with the AUTOBUILD routine of program suite PHENIX^59^, which resulted in a Fourier map that was used for manual model building with the COOT program^60^. The latter alternated with crystallographic refinement with PHENIX^61^ and BUSTER/TNT^62^ under inclusion of non-crystallographic symmetry restraints and translation/libration/screw-rotation refinement until completion of the model. The final refined model comprised residues A_1_-S_199_ and A_1_-L_200_ of astacin molecules A and C, respectively, *plus* a zinc cation each; segments A^25^-P^215^ + S^229^-E^258^ + R^364^-S^376^ and N^27^-P^215^ + S^229^-S^259^ + T^365^-A^375^ of hFB molecules B and D, respectively; and 31 solvent molecules (see Table 1 for the final model statistics). Residues N^37^ of molecules B and D had the glycan structure NAG(FUC)-NAG-BMA attached (for sugar moiety nomenclature, see the legend of Table 1). Residues N^136^ of molecules B and D showed only two and one NAG moieties attached, respectively. The quality of the final model was assessed with the wwPDB X-ray Structure Validation Service (https://validate.wwpdb.org ^63^).

### Proteolytic susceptibility of human and mouse fetuin-B

Both inhibitors were incubated with astacin in 150 mM sodium chloride, 50 mM Tris·HCl, pH 7.4. N-terminal sequencing by Edman degradation was performed by Proteome Factory AG (Berlin, Germany). Immunoblot analysis was performed as reported^35^ using a polyclonal hFB antibody as reported^50^ and a penta-His-antibody (Qiagen, Hilden, Germany). Cleaved hFB was isolated from the complex by astacin affinity chromatography. To this aim, astacin was covalently coupled to a HiTrap NHS-Activated HP affinity column (GE Life Sciences, Freiburg, Germany) according to the manufacturer’s specifications. Protein hFB was applied to the column and cleaved by addition of further astacin. The cleaved hFB was eluted by addition of 6 M urea, desalted and concentrated. The non-cleaved hFB control was treated similarly except for cleavage by astacin. The residual inhibitory capacities of both samples against astacin and human meprins α and β were determined.

### Inhibition assays *in vitro*

Inhibition of meprin α, meprin β and astacin by intact and astacin-cleaved hFB was determined *in vitro* in a Varioskan Flash 3001 spectral plate reader with SKANIT 2.4.3.RE software (ThermoFisher Scientific, Dreieich, Germany) with a fluorescence-based activity assay as previously described for mFB^36^. Enzyme concentrations were determined by absorbance at 280 nm (ε_astacin_ = 42,800 M^−1^ cm^−1^; ε_meprin α_ = 106,520 M^−1^ cm^−1^; ε_meprin β_ = 113,385 M^−1^ cm^−1^) and assays were performed in triplicate at 37 °C in 100 µl final volume, with 150 mM sodium chloride, 50 mM Tris·HCl, pH 7.4 and 0.01% Brij-35 as buffer. Cleavage reactions were initiated by the addition of 20–30 µM Ac-R-E(Edans)-D-R-Nle-V-G-D-D-P-Y-K(Dabcyl)-NH_2_ (Biosyntan, Berlin, Germany) for meprins α and β, and 170–180 µM Dansyl-P-K-R-P-W-V-OH (PANATecs, Heilbronn, Germany) for astacin, both predissolved in dimethyl sulfoxide (final concentration 0.4%). Initial velocities were recorded for at least 600 s (100 times for 100 ms at intervals of 15 s). Thereafter, 1.5 µl of proteinase K (Sigma-Aldrich) at 20 mg/ml or 1 µl astacin at 100 µM were added for complete substrate turnover. The latter was monitored and subsequently calculated using the formula v = [S] × m/ΔF, where [S] is the substrate concentration, m the [F/t] slope of initial linear substrate turnover, and ∆F the maximal fluorescence intensity corresponding to complete turnover. Kinetic parameters of inhibition (*K*_i_) were determined using Morrison’s equation^64^.

### Bioinformatics

Structure figures were prepared with the CHIMERA program^65^. Structure superimpositions were performed with SSM^66^ within COOT. Protein interfaces were analyzed with PISA^46^ (www.ebi.ac.uk/pdbe/pisa). The area of the interface of a complex was taken as half the sum of the buried surface areas of either molecule. Sequence identities were calculated with SIM with default parameters (https://web.expasy.org/cgi-bin/sim/sim.pl?prot). Signal peptides were predicted with SIGNALP v. 5.0 at http://www.cbs.dtu.dk/services/SignalP-5.0^67^. The final coordinates of the crayfish astacin·human fetuin-B complex are available from the PDB at www.rcsb.org (code 6SAZ).
